# Impact of Huisheng Oral Solution Combined with Immune Checkpoint Inhibitors and Chemotherapy in Patients with Stage III-IV Non-Small Cell Lung Cancer: A Retrospective Analysis

**DOI:** 10.7150/jca.116142

**Published:** 2025-07-28

**Authors:** Yifeng Bai, Jirui Wang, Linsheng Song, Honglin Hu, Lan Yang, Gang Feng, Jie Chen, Heling Peng, Shengkun Peng

**Affiliations:** 1 Department of Oncology, Sichuan Academy of Medical Sciences and Sichuan Provincial People's Hospital, University of Electronic Science and Technology of China, Chengdu, 610071, Sichuan Province, China.; 2 Department of Radiology, Sichuan Academy of Medical Sciences and Sichuan Provincial People's Hospital, University of Electronic Science and Technology of China, Chengdu, 610071, Sichuan Province, China.; 3 Department of Thoracic Surgery, Sichuan Provincial People's Hospital, University of Electronic Science and Technology of China, Chengdu, 610071, Sichuan Province, China.; 4 Department of Breast Surgery, Sichuan Academy of Medical Sciences and Sichuan Provincial People's Hospital, University of Electronic Science and Technology of China, Chengdu, 610071, Sichuan Province, China.; 5 Department of Medical Administration Division, Sichuan Provincial People's Hospital, University of Electronic Science and Technology of China, Chengdu, 610071, Sichuan Province, China.

**Keywords:** non-small cell lung cancer, immune checkpoint inhibitors, Huisheng oral solution, chemotherapy, immune-related adverse events

## Abstract

**Background:** To evaluate the impact of Huisheng Oral Solution (HSOS) in conjunction with immune checkpoint inhibitors (ICIs) and chemotherapy on patients with stage III-IV non-small cell lung cancer (NSCLC).

**Methods:** This retrospective study included patients with stage III-IV NSCLC who were treated at Sichuan Provincial People's Hospital from May 2018 to June 2021. Patients were categorized into two groups: the ICIs & Chemo Group and the ICIs & Chemo & HSOS Group, based on the therapies administered. The disease control rate (DCR), objective response rate (ORR), progression-free survival (PFS), overall survival (OS), and immune-related adverse events (irAEs) were assessed.

**Results:** A total of 185 patients were included, with 109 patients in ICIs & Chemo & HSOS Group. The ICIs & Chemo & HSOS Group exhibited significantly enhanced DCR (90.83% vs. 71.05%, p=0.001) compared to the ICIs & Chemo Group. The ORR was not statistically significant between the two groups (31.19% vs. 27.63%, p=0.628). Patients in the ICIs & Chemo & HSOS Group had significantly longer PFS (HR=0.47, 95% CI: 0.29-0.75, p<0.001) and OS (HR=0.58, 95% CI: 0.33-1.00, p=0.037) than the ICIs & Chemo Group. In terms of irAEs, nephrotoxicity (5.77% vs. 15.25%, p=0.044), checkpoint inhibitor-related pneumonitis (CIP) (2.75% vs. 11.84%, p=0.014), and cardiotoxicity (0% vs. 13.04%, p=0.026) were significantly lower in the ICIs & Chemo & HSOS Group.

**Conclusion:** The addition of HSOS to ICIs and chemotherapy may enhance DCR, PFS, and OS, while concurrently reducing irAEs in patients with stage III-IV NSCLC. These findings suggest that HSOS may serve as a promising adjunct to ICI-based therapies. Further prospective studies are warranted to validate these results.

## Introduction

Lung cancer remains one of the most prevalent and lethal malignancies worldwide, accounting for 11.4% of all cancer cases and 18.0% of all cancer-related deaths 1. Non-small cell lung cancer (NSCLC) constitutes approximately 85% of all lung cancer cases 2. In China, an estimated 800,000 individuals are diagnosed with lung cancer annually, with a 5-year overall survival (OS) rate of less than 20% 3. Since the 1990s, platinum-based doublet chemotherapy has served as the cornerstone of first-line treatment for advanced NSCLC. However, the efficacy of chemotherapy as a monotherapy remains limited 4, 5. Combination therapies incorporating other treatment modalities have become the standard of care, particularly for advanced-stage patients 6.

Recent advancements in immunotherapy, particularly with immune checkpoint inhibitors (ICIs), have revolutionized the treatment landscape for NSCLC 7. ICIs, including programmed death receptor-1 (PD-1) and programmed death-ligand 1 (PD-L1) inhibitors, are now widely used in the first- and second-line treatment of advanced NSCLC, as well as in the adjuvant treatment of locally advanced disease 8. Numerous clinical studies have demonstrated that ICIs enhance the immunosuppressive tumor microenvironment (TME) and potentiate the immune system's natural anti-tumor capabilities 9. When combined with other therapies, ICIs have been shown to increase tumor remission rates and reduce mortality compared to monotherapy. However, improvements in progression-free survival (PFS) and OS in second-line treatment and adjuvant settings remain inconsistent 10, 11. Despite their clinical efficacy, ICIs are associated with immune-related adverse events (irAEs), which can significantly impact multiple organ systems. Common irAEs include rash, colitis, hepatitis, myocarditis, endocrine dysfunction, and checkpoint inhibitor pneumonitis (CIP) 12. Severe irAEs, particularly grade 3-4 toxicities, are often managed with glucocorticoids and immunosuppressive agents, though these treatments have limited effectiveness and can introduce additional side effects 13.

Traditional Chinese Medicine (TCM) has a long history of complementing conventional cancer therapies by enhancing efficacy, improving tolerance, and mitigating adverse effects 14, 15. Huisheng Oral Solution (HSOS), a Chinese patent medicine derived from the classic Qing Dynasty prescription *Hua Zheng Hui Sheng Dan,* has demonstrated therapeutic potential in NSCLC^16^, ^17^. Produced by Chengdu Di'ao Group Tianfu Pharmaceutical Co., Ltd. (Approval number: Z20025042), HSOS has been shown to improve short-term efficacy, prolong survival, and reduce adverse reactions in NSCLC patients when used in combination with chemotherapy, radiotherapy, targeted therapy, or immunotherapy 16, 18, 19. Mechanistically, HSOS enhances immune function and modulates T lymphocyte subsets 20. Preclinical studies have further suggested that HSOS can inhibit PD-1/PD-L1 expression and the activation of related signaling pathways, potentially enhancing the efficacy of immunotherapy while reducing irAEs 21.

To date, clinical evidence on the therapeutic benefits of HSOS combined with ICIs and chemotherapy for advanced NSCLC remains limited. This study retrospectively analyzed the impact of this combination therapy in patients with stage III-IV NSCLC.

## Methods

### Study design and patients

This retrospective study analyzed data from patients with stage III-IV NSCLC who were treated in the Oncology Department of Sichuan Provincial People's Hospital between May 2018 and June 2021. This study was approved by the Institutional Review Board of Sichuan Provincial People's Hospital (Approval No.: EC Review (Research) No.11,202). As this study involved retrospective analysis using de-identified data, informed consent was not required. The inclusion criteria were as follows: (1) primary diagnosis of NSCLC confirmed by histopathology; (2) stage III-IV disease 22; (3) all patients received a combination of ICIs and chemotherapy; (4) at least one measurable lesion; (5) age ≥18 years; and (6) complete clinical data available. Exclusion criteria included: (1) mixed small-cell lung cancer and NSCLC histology; (2) prior exposure to PD-1/PD-L1 inhibitors; and (3) current or previous use of immunosuppressive medications. Patients were divided into two groups based on their therapies. The ICIs & Chemo group comprised patients who only received ICIs in combination with chemotherapy. The ICIs & Chemo & HSOS group included patients who received the same combination of ICIs and chemotherapy, with the addition of HSOS therapy. The ICIs used in both groups were as follows: Pembrolizumab (2 mg/kg, every 3 weeks), Sintilimab (200 mg, every 3 weeks), Camrelizumab (200 mg, every 3 weeks), Toripalimab (240 mg, every 3 weeks), and Tislelizumab (200 mg, every 3 weeks). Chemotherapy regimens included either paclitaxel or pemetrexed combined with platinum-based agents. Patients in the ICIs & Chemo & HSOS Group received 10 mL of HSOS orally three times daily. All patients underwent at least four treatment cycles, with each cycle lasting approximately 21 days.

### Outcome

The outcome measures included the objective response rate (ORR), disease control rate (DCR), OS, PFS, and immune-related adverse events (irAEs). Objective response was assessed using RECIST v1.1 criteria, and evaluated by investigators based on imaging studies obtained at intervals of approximately two immune cycles 23. According to the RECIST v1.1 criteria, Complete Response (CR) is defined as the disappearance of all target lesions, with no new lesions appearing. Partial Response (PR) is characterized by a reduction of at least 30% in the sum of the diameters of target lesions, using baseline measurements as a reference. Stable Disease (SD) refers to a condition where there is neither sufficient shrinkage to qualify for PR nor sufficient increase to meet the criteria for Progressive Disease (PD). Progressive Disease (PD) is defined as a 20% or greater increase in the sum of the diameters of target lesions, with an absolute increase of at least 5 mm or the appearance of new lesions. The ORR is calculated as the proportion of patients who achieve either CR or PR. The DCR was defined as the proportion of patients achieving CR, PR, or SD.

Follow-up was conducted through outpatient appointments and telephone consultations until October 2022. OS was defined as the time from treatment initiation to the date of death. Patients who were lost to follow-up or alive at the end of the study were censored at their last follow-up date. PFS was defined as the time from treatment initiation to disease progression or death. For patients who were lost to follow-up or had no documented progression, the cut-off time was the last follow-up date. According to previous studies on the impact of PD-1/PD-L1 inhibitors in small-cell lung cancer, the median PFS was around 7 to 8 months, and the median OS was around 18 to 19 months 24, 25. Therefore, poor prognosis was defined as the occurrence of events (death or progression) within 6 months, while good prognosis was defined as the absence of events (no death or progression) beyond 18 months in this study. The differences in prognosis between the two groups were analyzed.

The evaluation of immune-related toxicities included liver function (aspartate aminotransferase [AST] and alanine aminotransferase [ALT]), renal function (creatinine [CRE]), thyroid function (thyroid-stimulating hormone [TSH]), cardiac toxicity (myocardial enzyme levels), and CIP. The NCCN Guidelines: Management of Immunotherapy-Related Toxicities, Version 1.2020 26 were used to define the evaluation standards for hepatorenal toxicity, cardiotoxicity, and thyroid dysfunction. CIP and myocarditis were assessed using the Common Terminology Criteria for Adverse Events (CTCAE Version 4.03) 24 and were primarily determined by the lead clinical physician for each patient. The manifestations of CIP were categorized into four types: nonspecific interstitial pneumonia (NSIP), cryptogenic organizing pneumonia (COP), acute interstitial pneumonia/acute respiratory distress syndrome (AIP/ARDS), and hypersensitivity pneumonia (HP). The diagnostic criteria for myocardial infarction considered patients with normal high-sensitivity troponin levels (<1.5 ng/L) during baseline screening and a significant elevation in troponin levels following immunotherapy.

### Statistical analysis

All statistical analyses were performed using SPSS 24.0 (IBM Corp., Armonk, NY, USA) and GraphPad Prism 8.0 (GraphPad Software, San Diego, CA, USA). Quantitative data were described using medians and ranges, while categorical data were presented as counts (N) and percentages (frequencies). Comparisons of categorical variables were conducted using χ² tests. Kaplan-Meier survival curves were constructed to evaluate PFS and OS, and differences between groups were assessed using the log-rank test. Univariate and multivariate Cox proportional-hazards regression models were used to identify prognostic factors for PFS and OS. Statistical significance was defined as a two-sided p-value < 0.05.

## Results

### Baseline characteristics of the patients

A total of 185 patients with stage III-IV NSCLC were included in the study, with 76 patients in the ICIs & Chemo group and 109 patients in the ICIs & Chemo & HSOS group. No statistically significant differences were observed between the two groups regarding gender, age, pathological type, disease stage, PD-L1 expression, driver gene expression, or the choice of ICIs and antiangiogenic drugs (all p > 0.05). The baseline characteristics of the patients are summarized in **Table 1**.

### ORR and DCR

In the ICIs & chemotherapy group, no patients achieved CR, while 21 patients achieved PR, 33 had SD, and 22 experienced PD. In the ICIs & chemotherapy & HSOS group, similarly, no CR were observed. However, 34 patients achieved PR, 65 had SD, and 10 developed PD. The ORR in the ICIs & Chemo Group and the ICIs & Chemo & HSOS Group was not statistically significant (31.19% vs. 27.63%, p = 0.628). However, the DCR was significantly higher in the ICIs & Chemo & HSOS Group compared to the ICIs & Chemo Group (90.83% vs. 71.05%, p = 0.001). Further analysis of clinical factors revealed that patients with squamous cell carcinoma (42.11% vs. 21.10%, p = 0.003) or lobulation (32.64% vs. 8.70%, p = 0.024) were more likely to have a higher ORR. Driver gene expression was identified as an influencing factor for DCR (p = 0.004) **(Table 2)**.

### PFS and OS

**Figure 1:**
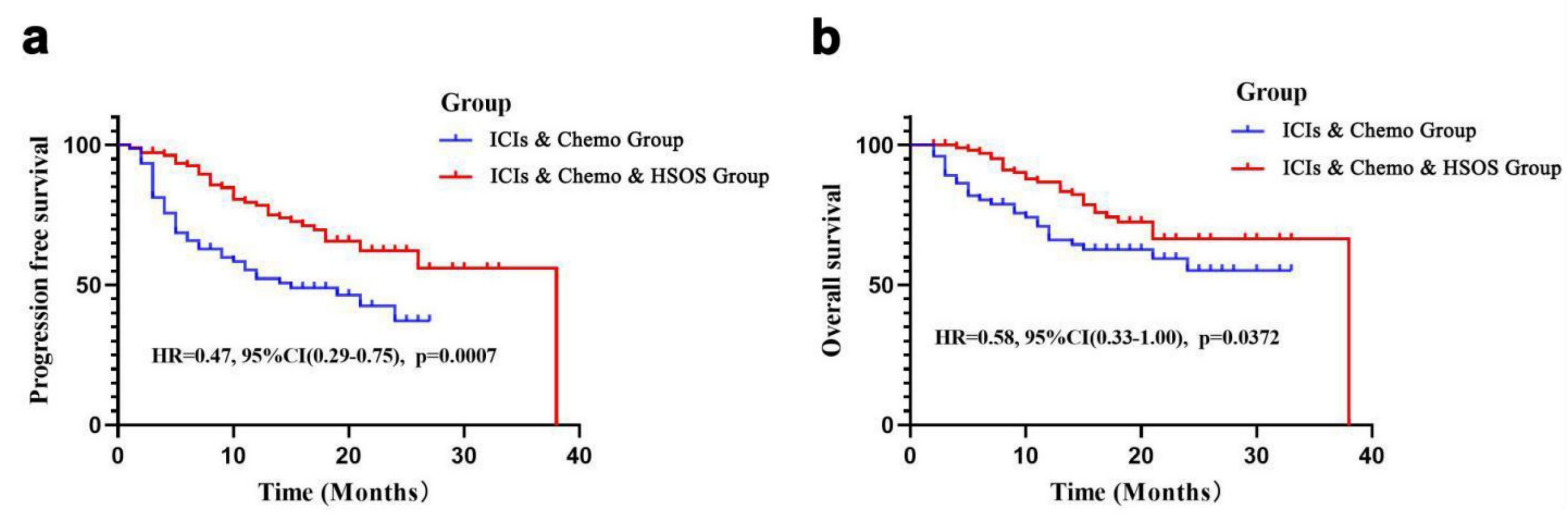
Survival analysis of PFS and OS in the two groups. a) PFS in the ICIs & Chemo Group and the ICIs & Chemo & HSOS Group; b) OS in thethe ICIs & Chemo Group and the ICIs & Chemo & HSOS Group.

The median follow-up time was 14 months (range: 2-33 months) for the ICIs & Chemo Group and 16 months (range: 2-38 months) for the ICIs & Chemo & HSOS Group. By the end of the follow-up period, disease progression (including death) had occurred in 39 patients in the ICIs & Chemo Group and 34 patients in the ICIs & Chemo & HSOS Group. The median PFS was 19 months in the ICIs & Chemo Group, while the estimated median PFS was not reached in the ICIs & Chemo & HSOS Group. Patients in the ICIs & Chemo & HSOS Group had significantly longer PFS compared to those in the ICIs & Chemo Group (HR = 0.47, 95% CI: 0.29-0.75, p < 0.001) **(Figure 1a)**. For OS, the estimated median OS was not reached in either group by the end of the follow-up. However, the ICIs & Chemo & HSOS Group exhibited better OS than the ICIs & Chemo Group (HR = 0.58, 95% CI: 0.33-1.00, p = 0.037) **(Figure 1b)**. Prognosis analysis based on predefined criteria showed that the ICIs & Chemo & HSOS Group had a higher proportion of patients with good prognosis compared to the ICIs & Chemo Group for both PFS (78.95% [30/38] vs. 41.86% [18/43], p < 0.001) and OS (92.11% [35/38] vs. 64.10% [25/39], p = 0.003), based on follow-up results.

### Prognostic factors for PFS and OS

In multivariate Cox regression analysis, the ICIs & Chemo & HSOS Group was independently associated with better PFS (HR = 0.44, 95% CI: 0.27-0.72, p = 0.001) and OS (HR = 0.48, 95% CI: 0.27-0.85, p = 0.011) **(Tables 3 and 4)**. Other prognostic factors for PFS included lymphangitis carcinomatosa (HR = 0.40, 95% CI: 0.21-0.76, p = 0.005), tracheobronchial sign (HR = 0.59, 95% CI: 0.35-0.99, p = 0.045), and hydrothorax (HR = 0.56, 95% CI: 0.33-0.96, p = 0.036) (**Figure S1 a, b, c**). Similarly, lymphangitis carcinomatosa (HR = 0.27, 95% CI: 0.13-0.64, p < 0.001), tracheobronchial sign (HR = 0.54, 95% CI: 0.30-0.99, p = 0.045), and hydrothorax (HR = 0.46, 95% CI: 0.25-0.83, p = 0.009) were associated with OS (**Figure S1 d, e, f**).

### Immune-related adverse events

There were no significant differences between the two groups in terms of hepatotoxicity (25.86% vs. 13.73%, p = 0.055) or endocrine toxicity (3.92% vs. 3.61%, p = 0.927). However, nephrotoxicity (5.77% vs. 15.25%, p = 0.044), CIP (2.75% vs. 11.84%, p = 0.014), and cardiotoxicity (TN) (0% vs. 13.04%, p = 0.026) was significantly lower in the ICIs & Chemo & HSOS Group compared to the ICIs & Chemo Group (**Table 5**). Further analysis indicated that lymph node metastasis, tracheobronchial signs, and chronic bronchitis were significant risk factors for the development of CIP (all p < 0.05, **Table S1**).

## Discussion

The results of this study suggest that the addition of HSOS to ICIs and chemotherapy may improve DCR, PFS, and OS, while reducing irAEs in patients with stage III-IV NSCLC. These findings underscore the clinical value of integrating HSOS into standard treatment regimens for advanced NSCLC, providing a promising strategy to enhance therapeutic efficacy and reduce irAEs.

This study demonstrated that the addition of HSOS, a Chinese patent medicine, to ICIs and chemotherapy might significantly improve clinical outcomes in patients with stage III-IV NSCLC. These findings are consistent with prior studies highlighting the advantages of combining HSOS for the treatment of advanced NSCLC 16, 17. The significant improvements in DCR, PFS, and OS observed in the ICIs & Chemo & HSOS Group compared to the ICIs & Chemo Group suggest that HSOS may act as a valuable adjunct by modulating the tumor microenvironment, reducing systemic inflammation, and enhancing immune function, as supported by preclinical studies 20, 21.

The reduction in irAEs observed in this study, including pulmonary toxicity, nephrotoxicity, and cardiotoxicity, underscores the potential role of HSOS in mitigating ICI-related toxicities. These findings align with the established role of Traditional Chinese Medicine (TCM) in reducing the adverse effects of cancer therapies 16, 18. Mechanistically, HSOS has been shown to regulate inflammatory and coagulation pathways, reduce fibrosis, and promote immune homeostasis 27. These effects make HSOS a promising candidate for addressing the challenges associated with ICI-induced toxicities, which are often managed with glucocorticoids and immunosuppressive agents that carry their own risks.

Compared to previous studies, the survival outcomes in our cohort are notably more favorable. For example, Wang et al. 28 reported a median PFS of 12.8 months for patients receiving ICIs and chemotherapy, while L. Paz-Ares et al. 29 and Martin Reck et al. 30 observed median PFS durations of 8.7 months and 6.7 months, respectively, with the same treatment approach. In contrast, our study demonstrated a median PFS of 19 months in the ICIs & Chemo Group, with an even longer PFS observed in the ICIs & Chemo & HSOS Group. The improved outcomes in our study may be attributed to the unique composition of HSOS, which includes immune-enhancing and anti-inflammatory components that complement the effects of ICIs. Additionally, differences in patient populations, and treatment durations may account for the observed discrepancies.

Our analysis also identified specific imaging and clinical features, such as malignant lymphangitis, tracheobronchial signs, and hydrothorax, as prognostic factors for worse outcomes. These findings are consistent with prior studies linking these features to advanced disease burden, distant metastasis, or impaired respiratory function 31. However, the role of these factors in predicting treatment response and toxicity in the context of HSOS requires further investigation.

While our results align broadly with the current literature, some differences should be noted. Studies evaluating ICIs alone or in combination with chemotherapy frequently report higher rates of severe irAEs, including CIP and myocarditis 12. The significantly lower rates of these toxicities in our study may reflect the protective effects of HSOS, which has been shown to inhibit PD-1/PD-L1 signaling pathways and modulate immune-related cytokines 27. These findings suggest that HSOS may not only enhance therapeutic efficacy but also improve the tolerability of ICIs, making it a promising addition to combination regimens. The therapeutic potential of HSOS is grounded in its complex composition, which includes 34 herbal and invertebrate-based ingredients. These components work synergistically to address the complex pathogenesis of lung cancer, which involves phlegm, blood stasis, blood toxicity, and deficiency syndromes. For instance, Trionycis Carapax and Leonuri Herba dissipate pathological masses, while aromatic herbs such as Chuanxiong Rhizoma, Angelica sinensis, and Cortex Cinnamomi Cassiae reduce turbidity and activate Qi to relieve pain. Blood-activating herbs, including Semen Persicae and Carthami Flos, promote circulation and resolve stasis, while immune-enhancing ingredients such as Ginseng and Rehmanniae Radix Praeparata improve systemic resistance and immunity.

Mechanistically, HSOS has been shown to regulate key pathways involved in tumor progression and immune modulation. It inhibits the Wnt/β-catenin pathway, reducing β-catenin and CyclinD1 expression to suppress tumor proliferation. It also modulates the miR-200b/ZEB-1 and TGF-β1-Smad3 pathways, alleviating EMT, a process closely associated with fibrosis and tumor metastasis 32. Additionally, HSOS reduces hypercoagulability and inflammation in tumor blood by lowering levels of tissue factor (TF), fibrinogen (FIB), and interleukin-6 (IL-6), while inhibiting angiogenesis-related factors such as CD44, metalloproteinase-2 (MMP-2), and vascular endothelial growth factor (VEGF) 33. These effects contribute to its anti-tumor, anti-inflammatory, and anti-metastatic properties, which likely underlie the observed improvements in DCR, PFS, and OS in this study.

Furthermore, HSOS enhances immune response by inhibiting PD-1/PD-L1 pathways, promoting macrophage function, and increasing the secretion of interleukin-12 (IL-12) and interleukin-18 (IL-18) 29. These effects lead to thymus and spleen enlargement and improved systemic immunity, which may explain the significant reduction in irAEs observed in our cohort. Clinical evidence also supports its ability to regulate serum levels of VEGF, IL-6, MMP-9, TNF-α, and TGF-β, further contributing to its efficacy in combination with ICIs 27.

Despite its promising findings, this study has several limitations. First, as a retrospective, single-center study, it is subject to inherent biases, including selection bias and unmeasured confounders. Additionally, the retrospective design of the study led to incomplete documentation of imaging characteristics for some patients. Consequently, the number of evaluable cases varied across different clinical parameters. Second, the follow-up period was relatively short, and many patients had not yet experienced disease progression or death by the end of the study, limiting the ability to estimate median PFS and OS. Third, certain key clinical characteristics, such as PD-L1 expression rates, tumor mutation burden, and driver gene mutations, were not comprehensively analyzed, which may have influenced treatment responses and survival outcomes. Additionally, the small sample size in certain subgroups, such as those receiving antiangiogenic drugs, limits the generalizability of the findings. Future research should include larger, multicenter, prospective studies with longer follow-up periods to validate these results and provide more robust conclusions.

## Conclusion

This study demonstrates that the combination of HSOS with ICIs and chemotherapy significantly improves clinical outcomes in patients with stage III-IV NSCLC. The addition of HSOS may be associated with higher DCR, prolonged PFS and OS, and a reduced incidence of irAEs, including nephrotoxicity, pulmonary toxicity and cardiotoxicity. These findings suggest that HSOS may serve as a promising adjunct to standard treatment regimens for advanced NSCLC. Further prospective studies are needed to confirm these results and to explore the underlying mechanisms by which HSOS modulates immune responses and reduces irAEs.

## Supplementary Material

Supplementary figure and table.

## Figures and Tables

**Table 1:** Basic clinical information of included patients (n, %)

	ICIs & Chemo Group (n=76)	ICIs & Chemo & HSOS Group (n=109)	p
Gender			0.447
Male	64 (84.21)	86 (78.90)	
Female	12 (15.79)	23 (21.10)	
Age (years)			0.375
<60	36 (47.37)	59 (54.13)	
≥60	40 (52.63)	50 (45.87)	
Pathological type			0.762
Adenocarcinoma	46 (60.53)	63 (57.80)	
Squamous cell carcinoma	30 (39.47)	46 (42.20)	
Staging of disease			0.152
IIIA	8 (10.53)	7 (6.42)	
IIIB	23 (30.26)	21 (21.10)	
IIIC	5 (6.58)	11 (10.09)	
IVA	24 (31.58)	51 (46.79)	
IVB	16 (21.05)	19 (17.43)	
PD-L1 expression			0.969
Negative	10 (13.16)	14 (12.84)	
Positive	9 (11.84)	15 (13.76)	
Unknown	57 (75.00)	80 (73.40)	
Driver gene expression			0.319
Negative	3 (3.95)	5 (4.59)	
Positive	2 (2.63)	9 (8.25)	
Unknown	71 (93.42)	95 (87.16)	
Antiangiogenic drugs			0.128
None	58 (76.32)	82 (75.23)	
Endostar	6 (7.89)	17 (15.60)	
Bevacuzumab	10 (13.16)	10 (9.17)	
Anlotinib	2 (2.63)	0 (0)	
ICIs			0.359
Sintilimab	23 (30.26)	26 (23.85)	
Camrelizumab	32 (42.11)	59 (54.13)	
Pembrolizumab	8 (10.53)	13 (11.93)	
Tislelizumab	11 (14.47)	8 (7.34)	
Toripalimab	2 (2.63)	3 (2.75)	

Abbreviations: ICIs: Immune checkpoint inhibitors; HSOS: Huisheng oral solution; PD-L1: Programmed death-ligand 1

**Table 2:** Influence of treatment and clinical features on the efficacy of solid tumors (n, %)

	CR	PR	SD	PD	ORR (%)	DCR (%)	p for ORR	p for DCR
Group							0.628	0.001
ICIs & Chemo Group	0	21	33	22	21 (27.63)	54 (71.05)		
ICIs & Chemo & HSOS Group	0	34	65	10	34 (31.19)	99 (90.83)		
Gender							0.099	0.329
Female	0	6	21	8	6 (17.14)	27 (77.14)		
Male	0	49	77	24	49 (32.67)	126 (84.00)		
Age (years)							0.261	0.848
<60	0	32	46	17	32 (33.68)	78 (82.11)		
≥60	0	23	52	15	23 (25.56)	75 (83.33)		
Staging of disease							0.168	0.204
IIIA	0	5	6	4	5 (33.33)	11 (73.33)		
IIIB	0	16	22	6	16 (36.36)	38 (86.36)		
IIIC	0	8	7	1	8 (50.00)	15 (93.75)		
IVA	0	19	45	11	19 (25.33)	64 (85.33)		
IVB	0	7	18	10	7 (20.00)	25 (71.43)		
Pathological type							0.003	0.117
Squamous cell carcinoma	0	32	35	9	32 (42.11)	67 (88.16)		
Adenocarcinoma	0	23	63	23	23 (21.10)	86 (78.9)		
PD-L1 expression							0.057	0.052
Negative	0	5	11	8	5 (20.83)	16 (66.67)		
Positive	0	3	16	5	3 (12.50)	19 (79.17)		
Unknown	0	47	71	19	47 (34.31)	118 (86.13)		
Driver gene expression							0.231	0.004
Negative	0	1	5	2	1 (12.50)	6 (75.00)		
Positive	0	1	4	6	1 (9.09)	5 (45.45)		
Unknown	0	53	89	24	53 (31.93)	142 (85.54)		
ICIs							0.924	0.035
Sintilimab	0	14	27	8	14 (28.57)	41 (83.67)		
Camrelizumab	0	27	52	12	27 (29.67)	79 (86.81)		
Pembrolizumab	0	5	8	8	5 (23.81)	13 (61.90)		
Tislelizumab	0	7	14	2	7 (36.84)	21 (110.53)		
Toripalimab	0	2	1	2	2 (40.00)	3 (60.00)		
Antiangiogenic drugs							0.568	0.679
None	0	44	70	26	44 (31.43)	114 (81.43)		
Endostar	0	6	15	2	6 (26.09)	21 (91.3)		
Bevacuzumab	0	4	12	4	4 (20.00)	16 (80.00)		
Anlotinib	0	1	1	0	1 (50.00)	2 (100.00)		
Lymph node condition							0.153	0.074
None	0	6	21	12	6 (15.38)	27 (69.23)		
1	0	10	14	4	10 (35.71)	24 (85.71)		
2	0	26	45	9	26 (32.50)	71 (88.75)		
3	0	7	10	3	7 (35.00)	17 (85.00)		
Airway spread							0.194	0.096
No	0	46	90	22	46 (31.08)	136 (91.89)		
Yes	0	3	10	6	3 (15.79)	13 (68.42)		
Pleural spread							0.567	0.643
No	0	38	64	22	38 (30.65)	102 (82.26)		
Yes	0	11	26	6	11 (25.58)	37 (86.05)		
Lobulation							0.024	0.073
No	0	2	14	7	2 (8.70)	16 (69.57)		
Yes	0	47	76	21	47 (32.64)	123 (85.42)		
Burr							0.877	0.67
No	0	18	33	9	18 (30.00)	51 (85.00)		
Short burr	0	24	43	15	24 (29.27)	67 (81.71)		
Long burr	0	0	2	1	0 (0.00)	2 (66.67)		
Short burr+Long burr	0	7	12	3	7 (31.82)	19 (86.36)		
Vacuoles							0.502	>0.999
No	0	48	89	28	48 (29.09)	137 (83.03)		
Yes	0	1	1	0	1 (50.00)	2 (100.00)		
Cavity							0.337	0.693
No	0	44	84	27	44 (28.39)	128 (82.58)		
Yes	0	5	6	1	5 (41.67)	11 (91.67)		
Vessel convergence sign							>0.999	0.684
No	0	26	47	16	26 (29.21)	73 (82.02)		
Yes	0	23	43	12	23 (29.49)	66 (84.62)		
Lymphangitis carcinomatosa							0.157	0.168
No	0	47	80	23	47 (31.33)	127 (84.67)		
Yes	0	2	10	5	2 (11.76)	12 (70.59)		
Pleural indentation							>0.999	0.83
No	0	18	32	11	18 (29.51)	50 (81.97)		
Yes	0	31	58	17	31 (29.25)	89 (83.96)		
Tracheobronchial sign							0.839	>0.999
No	0	39	69	22	39 (30.00)	108 (83.08)		
Yes	0	10	21	6	10 (27.03)	31 (83.78)		
Chronic bronchitis							0.603	>0.999
No	0	42	80	25	42 (28.57)	122 (82.99)		
Yes	0	7	10	3	7 (35.00)	17 (85.00)		
Tuberculosis							0.420	>0.999
No	0	46	87	27	46 (28.75)	133 (83.13)		
Yes	0	3	3	1	3 (42.86)	6 (85.71)		
Emphysema							0.861	0.085
No	0	30	53	22	30 (28.57)	83 (79.05)		
Yes	0	19	37	6	19 (30.65)	56 (90.32)		
Lung bullae							0.796	>0.999
No	0	44	78	25	44 (29.93)	122 (82.99)		
Yes	0	5	12	3	5 (25.00)	17 (85.00)		
Exudation							0.059	0.647
No	0	30	71	19	30 (25.00)	101 (84.17)		
Yes	0	19	19	9	19 (40.43)	38 (80.85)		
Hydrothorax							>0.999	>0.999
No	0	39	71	22	39 (29.55)	110 (83.33)		
Yes	0	10	19	6	10 (28.57)	29 (82.86)		

Abbreviations: CR: Complete response; PR: Partial response; SD: Stable disease; PD: Progressive disease; ORR: Objective response rate; DCR: Disease control rate

**Table 3:** Univariate and multivariable Cox regression analysis of PFS

	Univariate analysis		Multivariate analysis	
	HR (95%CI)	p	HR (95%CI)	p
Group (ICIs & Chemo & HSOS Group vs. ICIs & Chemo Group)	0.47 (0.29-0.75)	0.0007	0.44 (0.27-0.72)	0.001
Gender (Female vs Male)	0.92 (0.51-1.68)	0.784		
Age (<60 vs ≥60)	0.72 (0.45-1.14)	0.158		
Staging of disease (Ⅲ vs Ⅳ)	0.78 (0.48-1.25)	0.298		
Pathological type (Squamous cell carcinoma vs Adenocarcinoma)	0.74 (0.45-1.20)	0.218		
Lymph node condition (No vs Yes)	1.44 (0.84-2.45)	0.184		
Airway spread (No vs Yes)	0.66 (0.33-1.34)	0.25		
Pleural spread (No vs Yes)	1.58 (0.86-2.91)	0.14		
Lobulation (No vs Yes)	1.27 (0.65-2.50)	0.486		
Burr (No vs Yes)	1.40 (0.86-2.28)	0.177		
Vacuoles (No vs Yes)	0.90 (0.12-6.46)	0.913		
Cavity (No vs Yes)	1.60 (0.50-5.11)	0.426		
Vessel convergence sign (No vs Yes)	1.50 (0.92-2.46)	0.108		
Lymphangitis carcinomatosa (No vs Yes)	0.40 (0.21-0.76)	0.005	0.50 (0.25-0.97)	0.04
Pleural indentation (No vs Yes)	1.18 (0.72-1.93)	0.513		
Tracheobronchial sign (No vs Yes)	0.59 (0.35-0.99)	0.045	0.69 (0.40-1.20)	0.189
Chronic bronchitis (No vs Yes)	0.70 (0.36-1.38)	0.301		
Tuberculosis (No vs Yes)	1.32 (0.32-5.39)	0.702		
Emphysema (No vs Yes)	0.91 (0.56-1.49)	0.707		
Lung bullae (No vs Yes)	1.86 (0.75-4.64)	0.181		
Exudation (No vs Yes)	0.89 (0.52-1.50)	0.654		
Hydrothorax (No vs Yes)	0.56 (0.33-0.96)	0.036	0.57 (0.34-0.98)	0.018

Abbreviations: PFS: Progression-free survival; OS: Overall survival; HR: Hazard ratio

**Table 4:** Univariate and multivariable Cox regression analysis of OS

	Univariate analysis		Multivariate analysis	
	HR (95%CI)	p	HR (95%CI)	p
Group (ICIs & Chemo & HSOS Group vs. ICIs & Chemo Group)	0.58 (0.33-1.00)	0.0372	0.48 (0.27-0.85)	0.011
Gender (Female vs Male)	0.84 (0.41-1.72)	0.636		
Age (<60 vs ≥60)	0.53 (0.30-0.92)	0.025	0.58 (0.32-1.06)	0.076
Staging of disease (III vs IV)	0.85 (0.49-1.47)	0.558		
Pathological type (Squamous cell carcinoma vs Adenocarcinoma)	0.88 (0.50-1.54)	0.657		
Lymph node condition (No vs Yes)	1.17 (0.62-2.21)	0.625		
Airway spread (No vs Yes)	0.66 (0.29-1.46)	0.301		
Pleural spread (No vs Yes)	1.57 (0.79-3.15)	0.209		
Lobulation (No vs Yes)	0.89 (0.38-2.10)	0.795		
Burr (No vs Yes)	1.69 (0.96-2.96)	0.067		
Vacuoles (No vs Yes)	0.59 (0.08-4.30)	0.605		
Cavity (No vs Yes)	1.09 (0.34-3.52)	0.88		
Vessel convergence sign (No vs Yes)	1.44 (0.81-2.57)	0.211		
Lymphangitis carcinomatosa (No vs Yes)	0.27 (0.13-0.64)	<0.001	0.29 (0.13-0.61)	0.001
Pleural indentation (No vs Yes)	1.12 (0.63-1.99)	0.695		
Tracheobronchial sign (No vs Yes)	0.54 (0.30-0.99)	0.045	0.82 (0.43-1.57)	0.544
Chronic bronchitis (No vs Yes)	0.62 (0.29-1.33)	0.219		
Tuberculosis (No vs Yes)	0.88 (0.21-3.63)	0.859		
Emphysema (No vs Yes)	0.74 (0.42-1.30)	0.289		
Lung bullae (No vs Yes)	3.84 (0.93-15.80)	0.063		
Exudation (No vs Yes)	0.90 (0.49-1.65)	0.736		
Hydrothorax (No vs Yes)	0.46 (0.25-0.83)	0.009	0.42 (0.24-0.80)	0.007

**Table 5:** Incidence of irAEs (n, %)

Indexes	ICIs & Chemo Group	ICIs & Chemo & HSOS Group	p
Hepatotoxicity (AST and ALT)	15/58 (25.86)	14/102 (13.73)	0.055
Nephrotoxicity (BUN and CRE)	9/59 (15.25)	6 /104 (5.77)	0.044
Pulmonary toxicity (CIP)	9/76 (11.84)	3/109 (2.75)	0.014
Endocrine toxicity (TSH)	2/51 (3.92)	3/83 (3.61)	0.927
Cardiotoxicity (TN)	3 /23 (13.04)	0/52 (0)	0.026

Abbreviations: AST: Aspartate aminotransferase; ALT: Alanine aminotransferase; BUN: Blood urea nitrogen; CRE: Creatinine; TSH: Thyroid-stimulating hormone; TN: Troponin; CIP: Checkpoint inhibitor pneumonitis; irAEs: Immune-related adverse event
