# Supplementary material for: Impact of Huisheng Oral Solution Combined with Immune Checkpoint Inhibitors and Chemotherapy in Patients with Stage III-IV Non-Small Cell Lung Cancer: A Retrospective Analysis
Source: J Cancer. 2025 Jul 28;16(12):3579–88. doi: 10.7150/jca.116142 (PMC12435248; doi:10.7150/jca.116142)
Supplement: Supplementary file 1 — Supplementary figure and table. [file jcav16p3579s1.pdf]

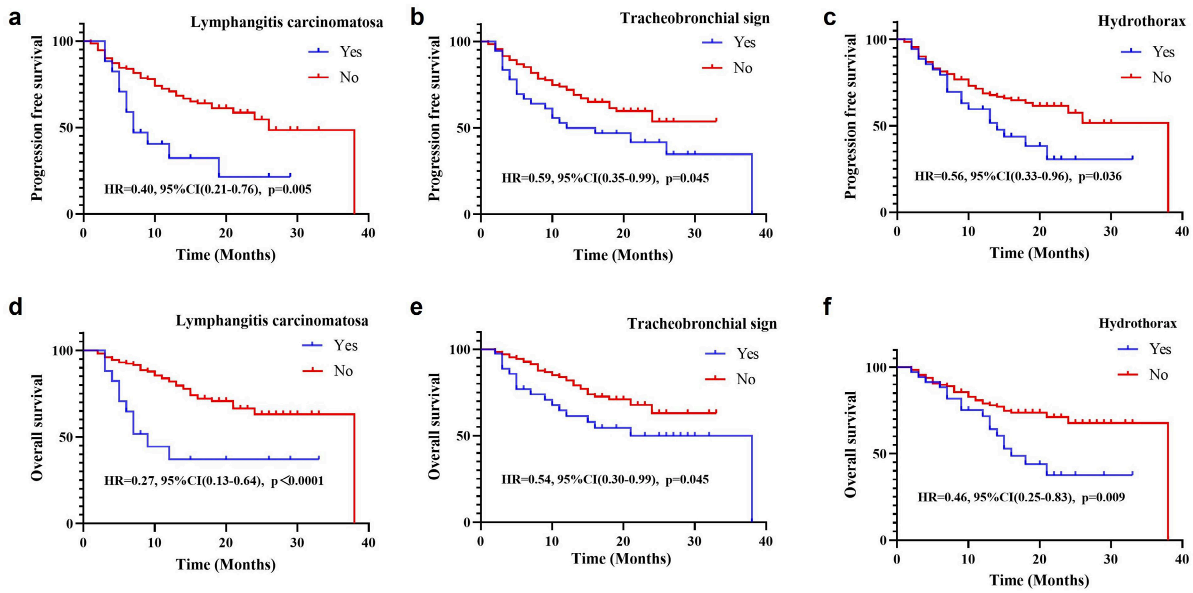

Figure S1. Survival analysis of PFS and OS in the two groups: a) PFS in patients with or without lymphangitis carcinomatosa; b) PFS in patients with or without tracheobronchial signs; c) PFS in patients with or without hydrothorax; d) OS in patients with or without lymphangitis carcinomatosa; e) OS in patients with or without tracheobronchial signs; f) OS in patients with or without hydrothorax.

Table S1. Relationship between CIP and other clinical factors of patients

**Variable**		**CIP**	**p**
**Gender**	Female (n=35)	0 (0)	0.127
	Male (n=150)	12 (8.00)	
**Age (years)**	＜60 (n=95)	5 (5.26)	0.559
	≥60 (n=90)	7 (7.78)	
**Staging of disease**	IIIA (n=15)	1 (6.67)	>0.999
	IIIB (n=44)	3 (6.82)	
	IIIC (n=16)	1 (6.25)	
	IVA (n=75)	5 (6.67)	
	IVB (n=35)	2 (5.71)	
**Pathological type**	Squamous cell carcinoma (n=76)	6 (7.90)	0.555
	Adenocarcinoma (n=109)	6 (5.50)	
**PD-L1 expression**	Negative (n=24)	1 (4.17)	0.384
	Positive (n=24)	3 (12.50)	
	Unknown (n=137)	8 (5.84)	
**Driver gene expression**	Negative (n=8)	0 (0)	>0.999
	Positive (n=11)	0 (0)	
	Unknown (n=166)	12 (7.23)	
**ICIs**	Sintilimab (n=49)	3 (6.12)	0.772
	Camrelizumab (n=91)	7 (7.70)	
	Pembrolizumab (n=21)	2 (9.520)	
	Tislelizumab (n=19)	0 (0)	
	Toripalimab (n=5)	0 (0)	

**Antiangiogenic drugs**	None (n=140)	11 (7.86)	0. 519
	Endostar (n=23)	0 (0)	
	Bevacuzumab (n=20)	1 (5.00)	
	Anlotinib (n=2)	0 (0)	
**Lymph node condition**	None (n=40)	1 (2.5)	0.019
	1 (n=33)	1 (3.0)	
	2 (n=88)	5 (5.7)	
	3 (n=24)	5 (20.8)	
**Airway spread**	None (n=163)	10(6.1)	0.597
	Yes (n=22)	2 (9.1)	
**Pleural spread**	None (n=138)	11 (8.0)	0.160
	Yes (n=47)	1 (2.1)	
**Lobulation**	None (n=28)	1 (3.6)	0.497
	Yes (n=157)	11 (7.0)	
**Burr**	None (n=68)	3 (4.4)	0.646
	Short burr (n=91)	8(8.8)	
	Long burr (n=3)	0(0)	
	Short burr+Long burr (n=23)	1 (4.3)	
**Vacuoles**	None (n=182)	12 (6.6)	0.646
	Yes (n=3)	0 (0)	
**Cavity**	None (n=171)	11 (6.4)	0.917
	Yes (n=14)	1 (7.1)	
**Vessel convergence sign**	None (n=99)	5 (5.1)	0.395
	Yes (n=86)	7 (8.1)	

**Lymphangitis carcinomatosa**	None (n=163)	9(5.5)	0.147
	Yes (n=22)	3(13.6)	
**Pleural indentation**	None (n=70)	3(4.3)	0.343
	Yes (n=115)	9 (7.8)	
**Tracheobronchial sign**	None (n=143)	4 (2.8)	<0.001
	Yes (n=42)	8 (19.0)	
**Chronic bronchitis**	None (n=161)	6 (3.7)	<0.001
	Yes (n=24)	6 (25.0)	
**Tuberculosis**	None (n=176)	12(6.8)	0.418
	Yes (n=9)	0 (0)	
**Emphysema**	None (n=108)	4(3.7)	0.069
	Yes (n=77)	8 (10.4)	
**Lung bullae**	None (n=159)	9(5.7)	0.259
	Yes (n=26)	3 (11.5)	
**Exudation**	None (n=130)	8 (6.2)	0.778
	Yes (n=55)	4 (7.3)	
**Hydrothorax**	None (n=145)	11 (7.6)	0.248
	Yes (n=40)	1 (2.5)	
